# Chemical Modification of Biomarkers through Accelerated Degradation: Implications for Ancient Plant Identification in Archaeo-Organic Residues

**DOI:** 10.3390/molecules27103331

**Published:** 2022-05-22

**Authors:** Barbara Huber, Daniel Giddings Vassão, Patrick Roberts, Yiming V. Wang, Thomas Larsen

**Affiliations:** 1 Department of Archaeology, Max Planck Institute for the Science of Human History, 07745 Jena, Germany; vassao@shh.mpg.de (D.G.V.); roberts@shh.mpg.de (P.R.); ywang@shh.mpg.de (Y.V.W.); 2 Department of Biochemistry, Max Planck Institute for Chemical Ecology, 07745 Jena, Germany; 3 School of Social Sciences, The University of Queensland, St Lucia, QLD 4072, Australia

**Keywords:** archaeological plant residues, residue identification, secondary metabolites, degradation experiment, catalysis, GC-MS, multivariate analysis

## Abstract

Biochemical and biomolecular archaeology is increasingly used to elucidate the consumption, use, origin, and trade of plants in the past. However, it can be challenging to use biomarkers to identify the taxonomic origin of archaeological plants due to limited knowledge of molecular survival and degradation for many key plant compounds in archaeological contexts. To gain a fundamental understanding of the chemical alterations associated with chemical degradation processes in ancient samples, we conducted accelerated degradation experiments with essential oil derived from cedar (*Cedrus atlantica*) exposed to materials commonly found in the archaeological record. Using GC-MS and multivariate analysis, we detected a total of 102 compounds across 19 treatments that were classified into three groups. The first group comprised compounds that were abundant in fresh cedar oil but would be unlikely to remain in ancient residues due to rapid degradation. The second group consisted of compounds that remained relatively stable or increased over time, which could be potential biomarkers for identifying cedar in archaeological residues. Compounds in the third group were absent in fresh cedar oil but were formed during specific experiments that could be indicative for certain storage conditions. These results show that caution is warranted for applying biomolecular profiles of fresh plants to ancient samples and that carefully designed accelerated degradation experiments can, at least in part, overcome this limitation.

## 1. Introduction

Biochemical and biomolecular analyses of plant residues from archaeological contexts is a rapidly expanding area of research [1,2,3,4,5,6,7,8]. Within this field, biomarkers are particularly useful indicators for ancient plant identification. Biomarker methodologies rely on the premise that particular biomolecular compounds or fingerprints found in archaeological samples can be linked to the known chemistry of modern plants [9,10]. With instrument advancement and increasing sensitivity of chromatographic and mass spectrometric methods, small molecules, especially secondary or specialized metabolites, including phenolics, alkaloids, benzenoids, and terpenes, are key targets for plant identification [11]. The structures of some of these biomolecules, e.g., terpenoids in plant resins or alkaloids in psychoactive plants, are in some cases, source diagnostic and thus, allow botanical genera and even species identification in comparison to modern plants [12,13,14]. The application of these methodologies in archaeological contexts has the potential to elucidate the use of material culture, consumption practices, and the origin and trade of certain plants in the past. Coniferous plant products, such as essential oils, resins, tars, and pitches derived from cedar and pine trees, for example, played an important role as they had multiple applications in the ancient world. These products were used as ingredients in medical remedies, cosmetics and perfumery, as waterproofing agents for containers, as glues, and in rituals, such as mummification [15,16,17,18]. However, using plant biomarkers for identifying the biological origin of archaeological residues comes with significant challenges. Numerous factors can substantially affect the biomolecular composition of ancient samples over time and might lead to incorrect plant identification if not considered [19]. It is also absolutely critical that the analyzed biomolecular fingerprints can be assumed to remain largely unaltered or at least predictably altered following their deposition.

A major challenge for identifying and interpreting ancient residues is the volatility of key diagnostic plant compounds, such sesquiterpenoids [14,20]. These volatile compounds can easily evaporate and change in archaeological samples over time, depending, for example, on environmental conditions and thermal fluctuations. The loss of these diagnostic markers can impede the ability to differentiate plant species. Moreover, compounds in archaeological samples can also undergo chemical alteration. Long-term deposition, weathering, biodegradation, combustion, light, oxidation, or reduction have all been demonstrated to lead to chemical transformations [21,22,23]. These processes may fragment compounds and form new derivatives that are generic for several plant species. For example, the phenolic compounds, vanillin and vanillic acid, occur naturally in vanilla extracts [24,25]. However, they are also common decomposition products of woody tissue and indicative of lignin pyrolysis [19,23,26]. Finally, even unfavorable chemical reactions can be greatly accelerated by catalysis, potentially leading to the modification of plant substances under the influence of organic or inorganic catalysts (see Box 1 for the definition of some chemical terms). In archaeological contexts this may happen when organic substances react with the surfaces or materials of archaeological containers and vessels, such as clay, gypsum, and different metals. These reactions can involve changes in covalent bonds, leading e.g., to condensations, addition, or removal of oxygenated groups, the intramolecular migration or replacement of functional groups (substitution), desaturation reactions, or cleavage of bonds (elimination) [27]. All these reactions and their potential to change the molecular composition of a given residue need to be considered in biomarker analysis and data interpretation. Nevertheless, although the effects of degradation on the chemical compositions of archaeological materials have been subject to many previous studies and experiments, most of these investigations have focused on the degradation of lipids [22,28,29,30] or wood/lignin [31,32,33,34,35].

Box 1 **Glossary for the definition of some chemical terms used in this study.**

**Activation energy:**
**Refers to the minimal quantity of energy required for the reactants to start a chemical reaction**.
**Aromatization:**
A chemical reaction in which an aromatic system is formed from a single nonaromatic precursor. Typically, aromatization is achieved by dehydrogenation of existing cyclic compounds.
**Catalyst:**
A substance that increases the rate of a reaction because it lowers the activation energy of the reactants. Catalytic specificity refers to the particular ability of a substance or closely related group of substances to catalyze a given type of chemical transformation. 
**Functional group:**
A structural unit of an atom or group of atoms within an organic compound that has its own characteristic property, regardless of the other atoms in the molecule.
**Redox:**
A chemical reaction where oxidation and reduction occur simultaneously. An atom is oxidized when it loses electrons and reduced when it gains electrons. Rust is the classic example of oxidation where the reduced iron metal (oxidation state 0) is oxidized to brown iron (oxidation state III) and oxides in the presence of catalysts, such as water, air, or an acid.
**Temperature:**
An increase in temperature will raise the average kinetic energy of the reactant molecules. As more molecules move faster, the number of molecules moving fast enough to react increases, which results in faster formation of products.

To date, there has been relatively little exploration of the potential and nature of secondary metabolite degradation to occur in plant residues, including resins, gums, tars, essential oils, spices, herbs, and psychoactive plant products. This is despite their widespread application in past medicinal, culinary, sanitary, cosmetic, ritual, and economic contexts [1,6,8,36,37,38,39,40,41,42,43,44]. Here, we investigate the transformation processes influencing the phytochemical composition of secondary metabolites from a selected plant source to gain a fundamental understanding of the chemical alterations associated with chemical degradation processes in ancient samples. We study these alterations by exposing modern reference samples to accelerated degradation treatments and subsequently analyzing them by gas chromatography coupled to mass spectrometry (GC-MS). By applying multivariate analysis to these complex data sets, we identify characteristic biomolecular profiles associated with the various degradation treatments. We use the oil of cedar trees (*Cedrus atlantica*) for the experiment, as coniferous plant products from the Pinaceae family are frequently found in organic residues from archaeological contexts, especially in artefacts from ancient Egypt [17,18,41,45,46,47,48,49]. Cedar essential oil is mainly composed of mono- and sesquiterpenes, with himachalenes as the main components [50]. We highlight the potential of the materials of archaeological containers to affect the chemical composition of their organic contents in diverse ways with some possible biomarkers disappearing from treated oils, while other derivatives are newly formed. Based on these results, we additionally propose valid archaeological biomarkers for identifying cedar plant products in archaeological residues. We argue that more comprehensive degradation studies are required to better understand the taphonomic pathways of secondary metabolites in different materials and geographical contexts and to develop more rigorous approaches to archaeological interpretation.

## 2. Experimental Design

In this study, nine different degradation experiments were designed to mimic the diagenetic effects of different reactions potentially catalyzed by archaeological materials and processes on compounds within ancient plant residues. As catalyst-driven alterations have thus far been insufficiently investigated in archaeological contexts, five different catalysts—bronze, copper, iron, clay (montmorillonite) and gypsum (CaSO_4_ 2H_2_O)—were selected to simulate archaeological materials. These are among the most common materials used to produce vessels that held organic substances in the past. To evaluate natural taphonomic processes, accelerated redox reactions were also separately induced using ammonium peroxodisulfate and sodium borohydride as oxidizing and reducing agents, respectively. Additionally, milder treatments that simulate these reactions naturally were examined, using air oxygen as an oxidant and nitrogen flushing to remove oxygen from the vial and create a non-oxidizing atmosphere (Table 1). To keep the number of treatments within a manageable range, this study leaves aside the influence of microbial activity as well the impact of exposure to light, which can cause free radical reactions and polymerization of compounds, such as terpenoids and phenolics. To promote different degrees of modification, each potential catalyst was investigated under both room temperature (RT) and at 80 °C (“high temperature”, HT) for seven-day periods with five replicates for each experiment. After mixing the catalysts with the oil, the mixtures sat in airtight 4 mL dram vials for the length of the experiments. A set of five untreated cedar oil samples served as the control treatment (see also *Materials and Methods* Section 4).

While we acknowledge that these treatments cannot exactly simulate long-term exposure to a burial environment or the storage within a particular vessel, the study has been designed to assess the major implications of different types of reactions and chemical catalyses for secondary metabolite degradation. As such, the results can provide broad insights into possible pathways of molecular transformation and loss in archaeological samples.

## 3. Results and Discussion

### 3.1. GC-MS Results and Multivariate Analyses

We detected a total of 102 compounds across all treatments after seven days of experimental incubation (Table 2 and Table S1). Of the 102 detected metabolites, 72 were present in the fresh cedar samples, and the remaining 30 were derivatives formed during the different degradation processes. The absolute peak areas were converted to relative abundances by dividing the area of each peak by the total peak area of a given sample.

The combination of temperature treatments and different potential catalysts simulating archaeological materials had varying effects on the degradation of secondary metabolites in fresh cedar tree oil and on the formation of derivatives. Taken together, we divided the observed compounds into three groups, based on the following criteria: The first group, G1, contains all compounds that were more abundant in the fresh cedar oil than in any of the degradation treatments (with the exception of a few compounds in N_2_ treatments and RedRT that were equal to fresh; see Tukey post-hoc multiple comparisons in Table S2). G1 comprised 20 compounds that decreased in the large majority of treatments relative to fresh samples, such as compound #32, whose relative abundance in the CuRT treatment was reduced to one-third compared to fresh. The second group, G2, is composed of 40 compounds that were present in fresh cedar oil, but whose relative abundance appeared to increase during exposure to a catalyst and heat, such as #55, which was 16 times more abundant in FeHT than in fresh oil. The other compound in this group tended to remain relatively stable and only slightly increased. The third and final group, G3, comprised 30 compounds that were absent in fresh cedar oil but present in one or several of the degradation treatments, as well as 12 compounds that were present in fresh cedar oil but in quantities that are barely detectable (<0.02% relative area). These compounds are the result of the chemical reactions during the experiment and are, therefore, likely to be present in archaeological samples. G3 compounds were more difficult to identify, and their relative abundances were generally lower than the G1 and G2 compounds.

**Figure 1:**
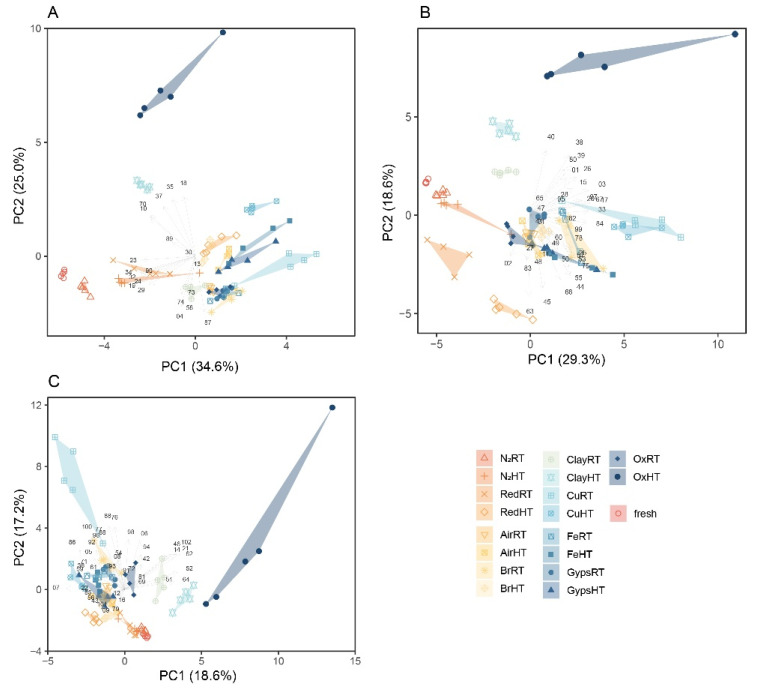
Principal component analyses (PCA) plots based on relative abundance values of each compound with each subplot (**A**–**C**) displaying the first two principal components for the compound groups G1, G2, and G3, respectively. Values in parentheses are the percentage variations accounted by each PC1 and PC2 axis, and the arrows represent the relative weightings of the independent variable, i.e., compound, for creating the PCA. See Supplementary Materials Figure S1 for a visualization of the first four principal components of each compound group.

The spread of the various treatments in the PCA plots across each of the three groups illustrates the contrasting effects of the various catalyst and temperature treatments on chemical profiles (Figure 1). Both treatments simulating a non-oxidizing atmosphere (N_2_RT and N_2_HT) and RedRT resembled the fresh samples in the G1 and G2 groups (Figure 1A,B). It is noticeable that both Cu treatments as well as the OxHT greatly influences chemical composition compared to that of the fresh samples and that the effect of temperature depends mostly on the catalyst used. For example, the effect of high temperature on the abundance of the G1 compounds stands out for Ox and Clay but less so for Air. For the G2 compounds, the effect of high temperature is particularly noticeable for Ox and Red, but not for Br, Cu, and N_2_ (except for one outlier sample) (Figure 1B). For the G3 compounds, the effect of temperature is comparatively less noticeable except for Ox (Figure 1C). Interestingly, RT causes more divergent chemical profiles than HT for Cu. The intra-treatment composition was largely consistent, i.e., samples within one treatment fell close to one another in the multivariate space except for the samples in the Ox- and ClayHT and CuRT treatments.

**Figure 2:**
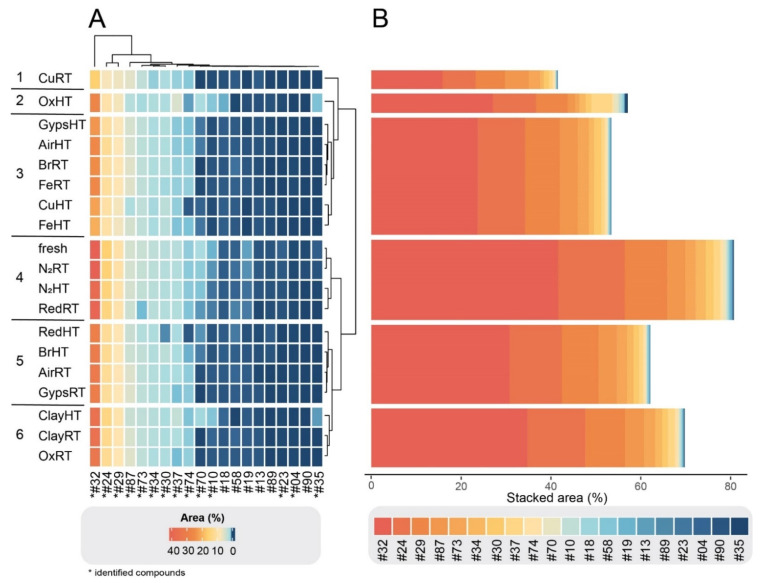
(**A**) Clustered heatmap of G1 compounds in which the color of a cell is proportional to its relative abundance (% area). The length of the branches represents the Euclidean distance or dissimilarity between clusters. The plot comprises 20 detected compounds that had reduced concentrations after the treatment. The horizontal bar plots (**B**) show stacked areas (%) of each of the degraded compounds (average relative abundance of all treatments per cluster).

**Figure 3:**
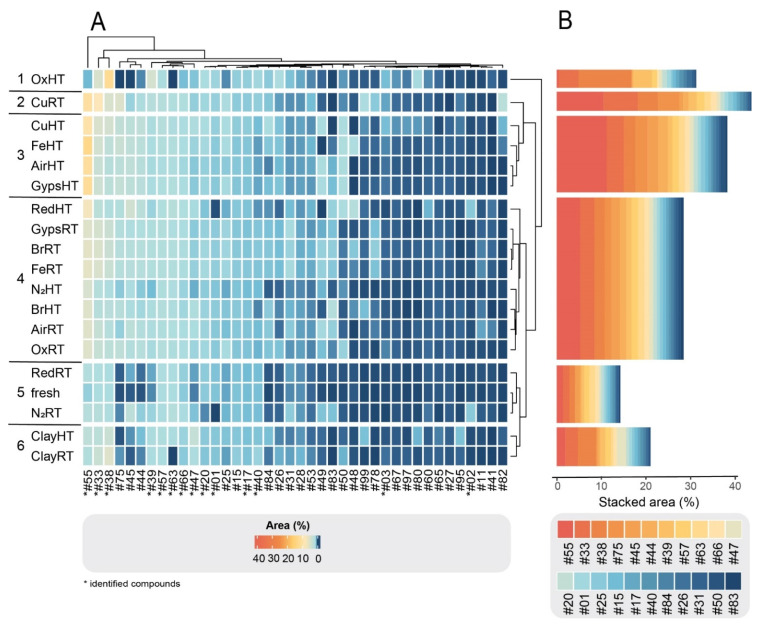
(**A**) Clustered heatmap of G2 compounds. The plot comprises 40 detected compounds that had increased concentrations after the treatment. The horizontal bar plots (**B**) show stacked areas (%) of each of the 20 most abundant degraded compounds.

**Figure 4:**
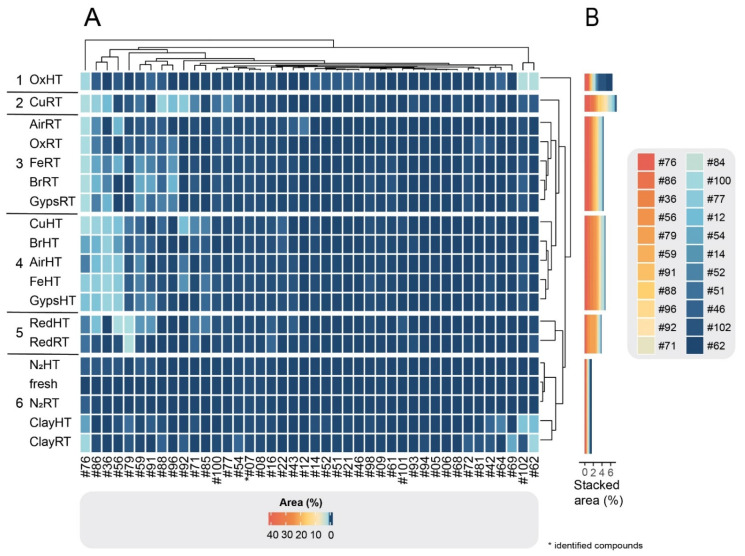
(**A**) Clustered heatmap of G3 compounds. The plot comprises 42 detected compounds that are absent or barely detectable in fresh cedar oil but present in one or several of the degradation treatments. The horizontal bar plots (**B**) show stacked areas (%) of each of the 20 most abundant degraded compounds.

Within each of the three compound groups, the treatments were divided into six clusters based on their similarities and dissimilarities in compound composition. As illustrated in the clustered heatmaps (Figure 2A, Figure 3A and Figure 4A), the color of a cell represents the relative abundance (% area) of each compound in each degradation experiment as well as the fresh sample. In all three groups, CuRT and OxHT always formed two separate clusters (1, 2) due to their high rate of degrading fresh compounds and forming of new ones. Both clusters showed major reductions in the relative abundance of compounds in G1 and displayed a strong increase in G2 and G3, demonstrating the significant effects of these treatments on the chemical compositions (Table S2).

In G1, fresh samples cluster with both N_2_ treatments and RedRT in cluster 4, showing that these treatments affected degradation of fresh G1 compounds minimally (Figure 2A). The clustering in G2 showed a similar distribution, where N_2_RT and RedRT were again grouped with the fresh sample (cluster 5; Figure 3A). This trend continued in G3 (Figure 4A) with slight variations in clustering patterns, resulting in both N_2_ and Clay treatments clustering with fresh samples (cluster 6) and Red treatments forming a distinct category (cluster 5) next to it.

Throughout all the groups, ClayRT and HT always fell into the same cluster (cluster 6), demonstrating that temperature impacts compounds in clay treatments minimally. More pronounced changes, however, were observed for gypsum and metals. Here, temperature played an important role, dividing these materials into two clusters in G3, placing the RT treatments in cluster 3 and HT treatments in cluster 4. Both clusters showed a substantial increase of certain compounds with very low concentrations or complete absences in the fresh samples, illustrating the impact of heat in catalyzing the formation of novel derivatives in these cases. Copper, iron, gypsum, and AirHT also clustered together in G2 (cluster 3). Again, this cluster was characterized by a high increase of compounds formed during degradation. However, the largest in G2, cluster 4, is composed of a mixture of RT and HT treatments, including both bronze treatments, Fe, Gyps, Air, and OxRT as well as Red and N_2_HT. In G1, the clusters containing the metals and gypsum (3 and 5) were also a mixture of HT and RT treatments. In all three groups, both Air treatments were always clustered together with gypsum and metal experiments. RT and HT Air are always divided into two different clusters, and their distributions resemble those of gypsum throughout all cluster groups.

To quantitatively assess changes in compound composition during degradation, we depicted the stacked areas of the most abundant compounds (for visual purposes only 20 compounds are depicted) for each group (Figure 2B, Figure 3B and Figure 4B). Each compound in the bar plots represents the average relative abundance of all treatments within each cluster. As expected from our criteria for dividing the 102 compounds into three groups, G1 cluster-4 containing the fresh treatment was by far the most abundant group of compounds, comprising stacked areas of 80%. In contrast, the two other clusters with the fresh treatment, G2 cluster-5 and G3 cluster-6, had low stacked areas of 15% and 2%, respectively (the remaining 3% is not visualized). The stacked areas also showed that the CuRT treatment followed by most of the HT treatments caused the greatest change in biomolecular profiles compared to the fresh oil.

When looking at specific compounds that played a larger diagnostic role among groups, the catalysts had a higher impact on the changes than temperature, as expected. In treatments with the reducing agent or clay, the differences between samples incubated at 80 °C and at RT were minimal for most compounds. For example, 4-acetyl-1-methylcyclohexene (#01, G2) was relatively stable throughout all treatments and in the fresh oils, with the exception of samples containing the reducing agent where this compound almost completely disappeared in both the RT (RedRT) and the HT (RedHT) treatments. The compound 3-cyclohexene-1-methanol, α,4-dimethyl- (#02, G2) can be used to illustrate the opposite effect. This compound had a very low concentration in the fresh sample, which did not change in the other treatments but is drastically increased in both RT and HT reduction treatments. However, some other compounds were also highly affected by temperature, particularly in experiments with the oxidation agent and the copper catalyst.

### 3.2. Compound Identification

The predominant compounds present in the fresh *Cedrus atlantica* oil were sesquiterpenes with the himachalene carbon skeleton generally found in the genus cedar [50,51,52,53,54], with the bicyclic sesquiterpene hydrocarbons α- γ- and β-himachalenes (#24, #29, #32) forming the largest peaks. Other constituents of the himachalane series, such as himachalene-1,4-diene (#30), α- and γ-dehydro-ar-himachalene (#33, #38), ar-himachalene (#39), oxidohimachalene (#47), (#55), and himachalol (#63) were detected in low amounts. Sesquiterpene ketones, such as atlantones, were also present in fresh oil.

Compared to the distributions of compounds from the degradation experiment, all sequiterpenoids from the himachalane series detected in fresh cedar oil were also present in the treated samples, although in variable proportions. Some almost completely disappeared, such as himachala-2,4-diene (#23), or were profoundly reduced in concentrations, such as himachalene-1,4-diene (#30) and β-himachalene (#32) (see G1 compounds). The latter drastically decreased in all samples with the Cu and Fe catalysts as well as in Ox, Gyps, and AirHT (G1, clusters 1–3). All the reduced himachalenes are compounds with nonconjugated double bonds, which makes them more sensitive to chemical alterations [40] and helps to explain the decrease of these constituents. Other compounds were only slightly reduced in abundance, such as the isomers α- and γ-himachalene (#24, #29), but remained relatively consistent throughout all experiments.

**Figure 5:**
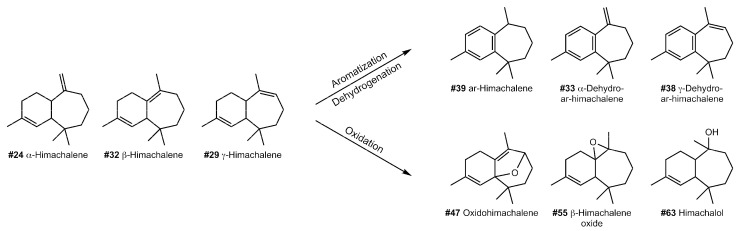
Aromatization, dehydrogenation, and oxidation of himachalenes in *Cedrus atlantica* essential oil.

The potentially most diagnostic compounds for ancient plant identification purposes are those that remain stable or relatively increased in peak areas after the treatments (i.e., G2 compounds), as they are more likely to be detected in ancient residues. Compared to the fresh oil, α- and γ-dehydro-ar-himachalenes (#33 and #38), ar-himachalene (#39) and oxidohimachalene (#47) increased in all treatments to varying extents. Most of them (#33, #38, #39) contain an aromatic functional group—a benzene ring, which gives the compound an increased thermodynamic and chemical stability (Figure 5). Again, the copper and the oxidizing agent had the strongest effect on the alteration of these compounds. The compound with the sharpest increase in most treatments is β-himachalene oxide (#55). The treatments of G2 clusters 2 and 3 resulted in an over 10-fold larger peak area than in the fresh sample. In contrast to other compounds, β-himachalene oxide also showed variations between RT and HT treatments, demonstrating the impact of temperature on the relative increase of the compound. The only experiment leading to a decrease in concentration of β-himachalene oxide was the oxidizing agent in combination with HT, whereas the same reactant at RT led to a stark increase of the peak.

Apart from the himachalenes, other sesquiterpenes, such as longifolene (#17) and vestitenone (#20), were also relatively stable and resistant to decay. Neither temperature nor catalysts seemed to severely affect the relative abundances of these constituents. By contrast, the ketone atlantone (#87), an abundant compound in the fresh cedar oil, varied in concentration after the experiments, depending on the catalysts as well as temperature. For example, the concentration was reduced severely in the Cu- and OxHT treatments but remained stable or decreased only slightly in the rest of the treatments. Another ketone, γ-atlantone (#70), almost completely disappeared following most treatments, except for the Clay- and OxHT treatment as well as both N_2_ treatments.

The most important degradation products that were absent or barely detectable in the fresh cedar samples (i.e., G3 compounds) are presented in Figure 4. These compounds result from of the chemical reactions during the experiment and might, therefore, likely be present in degraded archaeological samples. The clustering of groups shows a clear division based on the temperature and materials used in the treatment. However, the usefulness of those compounds in diagnosing a source material is limited, as most of them are difficult to detect and identify due to their low abundance. Generally, using degradation compounds as biomarkers requires knowledge of degradation pathways, which we still lack for the majority of plant species.

## 4. Materials and Methods

### 4.1. Materials

All solvents used in the experiments were of analytical grade and supplied by Sigma-Aldrich (Munich, Germany) from where calcium sulfate dihydrate (CaSO_4_ · 2H_2_O), sodium borohydride (NaBH_4_), and montmorillonite KSF (clay) were also purchased. Copper and iron powders (99%, -140 and -70 mesh, respectively) and ammonium peroxodisulfate (98+%) were purchased from Acros (Geel, Belgium), while bronze powder (Cu:Sn; 90:10 wt%, -100 mesh) was obtained from Alfa Aesar (Kandel, Germany). The essential oil of *Cedrus atlantica* was purchased from Florihana Distillerie (Caussols, France). The analytical standards (+)-α-longipinene, (+)-longifolene and (−)-isoledene were supplied by Sigma-Aldrich (Munich, Germany), α-terpineol, bisabolene (mixture of isomers), β-ionone, and ethanone,1-(4-methylphenyl)- by Thermo Fisher Scientific (Kandel, Germany) and 4-acetyl-1-methylcyclohexene by abcr (Karlsruhe, Germany).

### 4.2. Sample Preparation, Extraction and Analysis

For the experiment, 5 mg of each catalyst were weighed into 4 mL combusted (500 °C for 8 h) glass vials. Subsequently, 25 μL of essential oil of *Cedrus atlantica* were added to each vial. For each catalyst-oil-mixture, two experiments were prepared: (1) a ‘heat treatment’ where the glass vials were placed in a heating block for seven days at a constant 80 °C; and (2) a treatment of the mixtures at RT for seven days. For each treatment, five replicates were prepared, resulting in a total of 90 samples. All samples were tightly capped before the start of the experiment. For the experiment mimicking a non-oxidizing atmosphere, the vials were flushed with N_2_ for circa 5 s and quickly capped thereafter.

After letting all samples incubate for seven days, the samples from the HT treatment were removed from the heating block and centrifuged for 5 min at 500× *g*. Each of the samples was dissolved in 500 μL of CH_2_Cl_2_ and quickly (~5 s) mixed by vortexing. Essential oils and wood tars are in general completely soluble in CH_2_Cl_2_, which did not require additional extraction steps [41]. After letting samples rest for 5–10 min for settling of solid remains (catalysts), 10 μL of the extracts were taken out with glass capillaries into 1.5 mL combusted glass vials and diluted with 490 μL of CH_2_Cl_2_ for GC-MS analysis of the volatile fraction, which was carried out immediately afterwards. The five replicates of fresh, untreated oil were prepared in the same way as the treated samples.

GC-MS analysis was performed using an Agilent 8890 GC-System coupled to an Agilent 5977B GC/MSD. Chromatographic separation was achieved on a HP-5ms 60 m × 250 μm capillary column with a film thickness of 0.25 μm (Agilent, Waldbronn, Germany). The mass spectrometer was operated in electron impact (EI) mode at 70 eV with a scanning range from *m*/*z* 30 to 500 amu, and helium was used as a carrier gas. The GC oven temperature was held isothermally for 1 min at 60 °C, ramped to 150 °C at 30 °C/min and held for 1 min, increased at a rate of at 5 °C/min to 200 °C with a 1 min hold and then increased again at 15 °C/min to 320 °C with a final hold time of 1 min. The transfer line and source temperature were set at 250 °C and 230 °C, respectively. The total run time was 25 min with a solvent delay of 7 min. Injection volume was 1 μL with a split ratio of 5:1.

### 4.3. Data Pretreatment and Statistical Analysis

The Agilent MassHunter Qualitative Data Analysis software 10.0 was used for processing the GC-MS acquisition files. For peak integration, absolute peak area filters ≥1000 counts were used for exporting peak lists. Chromatographic peaks were identified based on comparison with retention times and mass spectra of analytical standards where available, by comparison to reference mass spectra in the NIST database (NIST 2.2), and with spectra reported in the literature. The peaks were aligned with the R package ‘CGalignR’ by setting the maximum distance for linear corrections to 0.06 and the minimum expected distance between peaks to 0.04 [55,56]. The chemical profiles (i.e., the relative distribution of compounds) of each replicate within each treatment were largely consistent except for two samples (AirHT.3 and FeHT.5). Since the cause of these very divergent profiles are unknown, we excluded these two outliers from the multivariate analyses. The peak areas of replicates from two metal RT treatments (BrRT replicates 2 and 5 and FeRT replicates 2 and 5) were approximately six time greater than the remaining replicates. However, we kept these four replicates in the multivariate analyses because their relative peak areas resembled the other replicates within their respective treatments. After manually inspecting the peak alignments, removing outlier peaks, and comparing peak areas across all samples, the data were converted to relative abundances by dividing the area of each peak by the total peak area of a given sample.

All statistical analyses were performed in R (version 4.0.1, R-Development-Core-Team, 2017-11-30) with R Studio interface version 1.3.959. The relative abundance of each peak was compared across treatments with a one-way ANOVA with Tukey’s HSD test. To identify similarities and differences among the treatments, we applied principal component analysis (PCA). PCA is an unsupervised technique that seeks to maximize variability among samples while reducing the number of dimensions. We rendered clustered heatmaps with the R package ‘ComplexHeatmap’ to reveal hierarchical clusters, which is a two-way display of a data matrix in which the colour of a cell is proportional to its position along a colour gradient. A group average was used for the cluster algorithms. The length of the branches represents the Euclidean distance or dissimilarity between clusters.

## 5. Conclusions

Our degradation experiments provide a means of better understanding the complexity of secondary metabolite transformation patterns of ancient plant residues. The careful consideration of catalyst-driven and natural chemical alterations when interpreting the chemistry of plant-derived compounds is expected to lead to a more accurate and rigorous source identification of residues attached to ancient vessels and containers from archaeological contexts. The compounds detected in the degradation experiments described herein were grouped into three categories:

1. G1 compounds disappeared or were decreased after seven days of incubation. If such stark declines were evident after only seven days, albeit under conditions that accelerate degradation, it can be assumed that they are very unlikely to remain in ancient residues, deposited for centuries or millennia. These compounds should, therefore, not be considered as diagnostic biomarkers for ancient plants and plant products under most conditions. Perhaps as importantly, their absence in archaeological samples cannot be considered as useful evidence for the absence of certain plants or for the identification of certain plants over other possible candidates.
2. G2 compounds remained relatively stable or increased over time, particularly oxidized and dehydrogenated compounds that have lost even numbers of hydrogen atoms to form double bonds, which generally makes them more stable and conceivably likely to remain in archaeological samples. These compounds might therefore be considered as more valid biomarkers aiding in the identification of archaeological residues.
3. G3 compounds were not present in fresh cedar oil but formed during specific experiments and are indicative of certain catalysts/storage materials. These compounds could, therefore, also be possible biomarkers for identifying plant materials in archaeological samples provided that these compounds can be identified. In our study, we were only able to securely identify one of the 42 G3 compounds. However, unknown compounds can still provide information regarding the processes involved in the preparation of plant-based products in the past. For example, compound #76 was relatively high in RT treatments in combination with Cu, Fe, and Br, showing that a high abundance of this compound could be indicative for the contact of cedar residues with metal vessels. Concomitantly, compound #79 appeared in high abundance in both reduction treatments, indicating a reduction process.

While the main purpose of this experiment was to raise awareness of the pitfalls and necessary considerations when using metabolic biomarkers from archaeological specimens for plant identification, it also led us to find robust archaeological biomarkers for the genus *Cedrus*. In general, the himachalenes appear to be good biomarkers for cedar species, as they rarely appear in other plants. We propose α- and γ-dehydro-ar-himachalene (#33 and #38), ar-himachalene (#39), oxidohimachalene (#47), and particularly β-himachalene oxide (#55) as promising molecular markers for identifying archaeological cedar samples, as they increase after redox and catalyst-driven reactions (see G2). Moreover, α-himachalene (#24), himachalol (#63), and allohimachalol (#66) also appear to remain relatively stable. Notably, β -himachalene (#32), the largest component of modern cedar oil, was rapidly reduced in all experiments. This reduction was detected after only seven days; hence, this compound most likely will not be well-preserved in archaeological samples. Similarly, himachala-2,4-diene (#23) and himachalene-1,4-diene (#30) should only be considered as archaeological biomarkers under very limited conditions (see G1). Longifolene (#17) and vestitenone (#20) have proven to be relatively resistant to decay.

Our findings can be compared to results from analyses of archaeological plant residues which were identified as plant products from cedar [41] where dehydrogenated analogues of sesquiterpenoids from himachalenes (e.g., #033 and #038) were also detected, while G1 himachalenes were absent. The similarities between archaeological findings and our accelerated degradation experiments underline the potential for expanding accelerated degradation studies to terpenoids from other plants, as well as to compounds belonging to other natural product classes. It is important to note that degradation conditions can be designed and refined to resemble particular archaeological settings in terms of the catalysts used but also that there is a scope for investigating how biotic conditions, such as bacterial or fungal degradation as well as exposure to light, alter plant metabolic profiles in the archaeological record. These types of data can serve as a cautionary note when identifying plants in ancient samples through comparison with modern materials and can help to avoid misidentification of materials based on common or unstable molecules.

## Figures and Tables

**Table 1:** Catalysts used to simulate metabolite reactions with organic compounds caused by archaeological materials (clay, gypsum, bronze, copper, and iron) or redox processes (oxidation, reduction).

Catalyst	Simulated Archaeological Material or Natural Reactions	Sample Code for Room RT and HT Treatments
Montmorillonite KSF clay (SiO_2_, Al_2_O_3_, H_2_SO, Fe_2_O_3_, CaO, MgO)	Clay	ClayRT, ClayHT
Calcium sulfate dihydrate (CaSO_4_ 2H_2_O)	Gypsum (alabaster)	GypsRT, GypsHT
Bronze powder (Cu:Sn, 90:10)	Bronze	BrRT, BrHT
Copper powder (Cu, 106 μm)	Copper	CuRT, CuHT
Iron powder (Fe, <212 μm)	Iron	FeRT, FeHT
Sodium borohydride (NaBH_4_)	Reduction	RedRT, RedHT
Flushing with N_2_	Non-oxidizing atmosphere	N_2_RT, N_2_HT
Ammoniumperoxodisulfat ((NH_4_)2S_2_O_8_)	Oxidation	OxRT, OxHT
Air	Oxidation	AirRT, AirHT

**Table 2:** Identified compounds by GC-MS analysis in the degradation experiments as well as in the fresh samples (29 out of 102). The remaining compounds could not be securely identified. Compounds displayed in bold were confirmed with analytical standards or had a match factor of >900. Compound names given in italic had a match factor >800.

ID	*t*_r_	Compound Identification
#01	8.28	**4-Acetyl-1-methylcyclohexene**
#02	8.46	**3-Cyclohexene-1-methanol, α,4-dimethyl-**
#03	8.94	**Ethanone, 1-(4-methylphenyl)-**
#04	8.99	**α-Terpineol**
#07	11.48	**α-Longipinene**
#10	11.90	*Isolongifolene, 4,5-dehydro-*
#17	12.52	**Longifolene**
#20	12.80	**Vestitenone**
#23	13.07	**Himachala-2,4-diene**
#24	13.24	**α-Himachalene**
#29	13.70	**γ-Himachalene**
#30	13.77	**Himachala-1,4-diene**
#32	14.09	**β-Himachalene**
#33	14.25	**α-Dehydro-ar-himachalene**
#34	14.31	**δ-Cadinene**
#35	14.39	**Calamene** (cis/trans?)
#37	14.49	**α-Bisabolene**
#38	14.58	**γ-Dehydro-ar-himachalene**
#39	14.72	**ar-Himachalene**
#40	14.80	*Calacorene (α/β?)*
#47	15.47	**Oxidohimachalene**
#55	16.32	**β-Himachalene oxide**
#57	16.51	*Epicubenol*
#63	17.03	**Himachalol**
#66	17.26	**Allohimachalol**
#70	17.48	*γ-Atlantone (E/Z?)*
#73	17.70	**2,2,6-Trimethyl-6-(4-methylcyclohex-3-en-1-yl)dihydro-2H-pyran-4(3H)-one**
#74	17.80	**α-Atlantone**
#87	18.79	**Atlantone**

## Data Availability

Data supporting reported results can be found in the Supplementary Materials and be downloaded at: https://www.mdpi.com/article/10.3390/molecules27103331/s1.

## Supplementary Materials

The following supporting information can be downloaded at: https://www.mdpi.com/article/10.3390/molecules27103331/s1, Table S1: Absolute peak areas of all samples; Table S2: One-way ANOVA along with the Tukey post-hoc multiple comparisons; Figures S1: PCA plots displaying the first four principal components of each of the G1, G2, and G3 compound groups.

## References

[1] Zimmermann M., Brownstein K.J., Pantoja Díaz L., Ancona Aragón I., Hutson S., Kidder B., Tushingham S., Gang D.R. (2021). Metabolomics-Based Analysis of Miniature Flask Contents Identifies Tobacco Mixture Use among the Ancient Maya. Sci. Rep..

[2] Brockbals L., Habicht M., Hajdas I., Galassi F.M., Rühli F.J., Kraemer T. (2018). Untargeted Metabolomics-like Screening Approach for Chemical Characterization and Differentiation of Canopic Jar and Mummy Samples from Ancient Egypt Using GC-High Resolution MS. Analyst.

[3] Dunne J., Mercuri A.M., Evershed R.P., Bruni S., di Lernia S. (2016). Earliest Direct Evidence of Plant Processing in Prehistoric Saharan Pottery. Nat. Plants.

[4] Hendy J., Colonese A.C., Franz I., Fernandes R., Fischer R., Orton D., Lucquin A., Spindler L., Anvari J., Stroud E. (2018). Ancient Proteins from Ceramic Vessels at Çatalhöyük West Reveal the Hidden Cuisine of Early Farmers. Nat. Commun..

[5] Scott A., Power R.C., Altmann-Wendling V., Artzy M., Martin M.A.S., Eisenmann S., Hagan R., Salazar-García D.C., Salmon Y., Yegorov D. (2020). Exotic Foods Reveal Contact between South Asia and the Near East during the Second Millennium BCE. Proc. Natl. Acad. Sci. USA.

[6] Riesmeier M., Keute J., Veall M.-A., Borschneck D., Stevenson A., Garnett A., Williams A., Ragan M., Devièse T. (2022). Recipes of Ancient Egyptian Kohls More Diverse than Previously Thought. Sci. Rep..

[7] Miller M.J., Whelton H.L., Swift J.A., Maline S., Hammann S., Cramp L.J.E., McCleary A., Taylor G., Vacca K., Becks F. (2020). Interpreting Ancient Food Practices: Stable Isotope and Molecular Analyses of Visible and Absorbed Residues from a Year-Long Cooking Experiment. Sci. Rep..

[8] Huber B., Larsen T., Spengler R.N., Boivin N. (2022). How to Use Modern Science to Reconstruct Ancient Scents. Nature Human Behaviour.

[9] Evershed R.P. (2008). Organic Residue Analysis in Archaeology: The Archaeological Biomarker Revolution. Archaeometry.

[10] Evershed R.P. (1993). Biomolecular Archaeology and Lipids. World Archaeol..

[11] Arimura G., Maffei M. (2021). Plant. Specialized Metabolism: Genomics, Biochemistry, and Biological Functions.

[12] Paul M., Brüning G., Bergmann J., Jauch J. (2012). A Thin-Layer Chromatography Method for the Identification of Three Different Olibanum Resins (*Boswellia Serrata*, *Boswellia Papyrifera* and *Boswellia Carterii*, Respectively, *Boswellia Sacra*): Thin-Layer Chromatography Identification Of Olibanum. Phytochem. Anal..

[13] Ribechini E., Raffaelli M., Colombini M.P., Avanzini A. (2008). Botanical and Chemical Characterization of Frankincense Resin from Dhofar. A port in Arabia between Rome and the Indian Ocean (3rd C. BC–5th C. AD).

[14] Salomé-Abarca L.F., van der Pas J., Kim H.K., van Uffelen G.A., Klinkhamer P.G.L., Choi Y.H. (2018). Metabolic Discrimination of Pine Resins Using Multiple Analytical Platforms. Phytochemistry.

[15] Degano I., Soriano S., Villa P., Pollarolo L., Lucejko J.J., Jacobs Z., Douka K., Vitagliano S., Tozzi C. (2019). Hafting of Middle Paleolithic Tools in Latium (Central Italy): New Data from Fossellone and Sant’Agostino Caves. PLoS ONE.

[16] Orengo H.A., Palet J.M., Ejarque A., Miras Y., Riera S. (2013). Pitch Production during the Roman Period: An Intensive Mountain Industry for a Globalised Economy?. Antiquity.

[17] Buckley S.A., Clark K.A., Evershed R.P. (2004). Complex Organic Chemical Balms of Pharaonic Animal Mummies. Nature.

[18] Fulcher K., Serpico M., Taylor J.H., Stacey R. (2021). Molecular Analysis of Black Coatings and Anointing Fluids from Ancient Egyptian Coffins, Mummy Cases, and Funerary Objects. Proc. Natl. Acad. Sci. USA.

[19] Whelton H.L., Hammann S., Cramp L.J.E., Dunne J., Roffet-Salque M., Evershed R.P. (2021). A Call for Caution in the Analysis of Lipids and Other Small Biomolecules from Archaeological Contexts. J. Archaeol. Sci..

[20] Capetti F., Rubiolo P., Bicchi C., Marengo A., Sgorbini B., Cagliero C. (2020). Exploiting the Versatility of Vacuum-assisted Headspace Solid-phase Microextraction in Combination with the Selectivity of Ionic Liquid-based GC Stationary Phases to Discriminate Boswellia Spp. Resins through Their Volatile and Semivolatile Fractions. J. Sep. Sci..

[21] Gelbrich J., Mai C., Militz H. (2008). Chemical Changes in Wood Degraded by Bacteria. Int. Biodeterior. Biodegrad..

[22] Malainey M.E., Przybylski R., Sherriff B.L. (1999). The Effects of Thermal and Oxidative Degradation on the Fatty Acid Composition of Food Plants and Animals of Western Canada: Implications for the Identification of Archaeological Vessel Residues. J. Archaeol. Sci..

[23] Tamburini D., Łucejko J., Ribechini E., Colombini M.P. (2016). New Markers of Natural and Anthropogenic Chemical Alteration of Archaeological Lignin Revealed by in Situ Pyrolysis/Silylation-Gas Chromatography mass Spectrometry. J. Anal. Appl. Pyrolysis.

[24] Brunschwig C., Collard F.X., Bianchini J.-P., Raharivelomanana P. (2009). Evaluation of Chemical Variability of Cured Vanilla Beans (*Vanilla tahitensis* and *Vanilla planifolia*). Nat. Prod. Commun..

[25] Linares V., Adams M.J., Cradic M.S., Finkelstein I., Lipschits O., Martin M.A.S., Neumann R., Stockhammer P.W., Gadot Y. (2019). First Evidence for Vanillin in the Old World: Its Use as Mortuary Offering in Middle Bronze Canaan. J. Archaeol. Sci. Rep..

[26] Lesage-Meessen L., Delattre M., Haon M., Thibault J.-F., Ceccaldi B.C., Brunerie P., Asther M. (1996). A Two-Step Bioconversion Process for Vanillin Production from Ferulic Acid Combining Aspergillus Niger and Pycnoporus Cinnabarinus. J. Biotechnol..

[27] Duce C., Orsini S., Spepi A., Colombini M.P., Tiné M.R., Ribechini E. (2015). Thermal Degradation Chemistry of Archaeological Pine Pitch Containing Beeswax as an Additive. J. Anal. Appl. Pyrolysis.

[28] Dudd S.N., Regert M., Evershed R.P. (1998). Assessing Microbial Lipid Contributions during Laboratory Degradations of Fats and Oils and Pure Triacylglycerols Absorbed in Ceramic Potsherds. Org. Geochem..

[29] Evershed R.P., Dudd S.N., Copley M.S., Berstan R., Stott A.W., Mottram H., Buckley S.A., Crossman Z. (2002). Chemistry of Archaeological Animal Fats. Acc. Chem. Res..

[30] Hammann S., Cramp L.J.E., Whittle M., Evershed R.P. (2018). Cholesterol Degradation in Archaeological Pottery Mediated by Fired Clay and Fatty Acid Pro-Oxidants. Tetrahedron Lett..

[31] Blanchette R.A. (2000). A Review of Microbial Deterioration Found in Archaeological Wood from Different Environments. Int. Biodeterior. Biodegrad..

[32] Colombini M.P., Lucejko J.J., Modugno F., Orlandi M., Tolppa E.-L., Zoia L. (2009). A Multi-Analytical Study of Degradation of Lignin in Archaeological Waterlogged Wood. Talanta.

[33] Gjelstrup Björdal C. (2012). Microbial Degradation of Waterlogged Archaeological Wood. J. Cult. Herit..

[34] Huisman D.J., Manders M.R., Kretschmar E.I., Klaassen R.K.W.M., Lamersdorf N. (2008). Burial Conditions and Wood Degradation at Archaeological Sites in the Netherlands. Int. Biodeterior. Biodegrad..

[35] Łucejko J.J., Modugno F., Ribechini E., Tamburini D., Colombini M.P. (2015). Analytical Instrumental Techniques to Study Archaeological Wood Degradation. Appl. Spectrosc. Rev..

[36] Devièse T., Ribechini E., Castex D., Stuart B., Regert M., Colombini M.P. (2017). A Multi-Analytical Approach Using FTIR, GC/MS and Py-GC/MS Revealed Early Evidence of Embalming Practices in Roman Catacombs. Microchem. J..

[37] Brettell R.C., Stern B., Reifarth N., Heron C. (2014). The ‘Semblance of Immortality’? Resinous Materials and Mortuary Rites in Roman Britain. Archaeometry.

[38] Giachi G., Pallecchi P., Romualdi A., Ribechini E., Lucejko J.J., Colombini M.P., Mariotti Lippi M. (2013). Ingredients of a 2,000-y-Old Medicine Revealed by Chemical, Mineralogical, and Botanical Investigations. Proc. Natl. Acad. Sci. USA.

[39] Regert M., Devièse T., Le Hô A.-S., Rougeulle A. (2008). Reconstructing Ancient Yemeni Commercial Routes during the Middle Ages Using Structural Characterization of Terpenoid Resins. Archaeometry.

[40] Ren M., Tang Z., Wu X., Spengler R., Jiang H., Yang Y., Boivin N. (2019). The Origins of Cannabis Smoking: Chemical Residue Evidence from the First Millennium BCE in the Pamirs. Sci. Adv..

[41] Sarret M., Adam P., Schaeffer P., Ebert Q., Perthuison J., Pierrat-Bonnefois G. (2017). Organic Substances from Egyptian Jars of the Early Dynastic Period (3100–2700 BCE): Mode of Preparation, Alteration Processes and Botanical (Re)Assessment of “Cedrium”. J. Archaeol. Sci. Rep..

[42] Serpico M., White R. (2000). The Botanical Identity and Transport of Incense during the Egyptian New Kingdom. Antiquity.

[43] Stern B., Heron C., Corr L., Serpico M., Bourriau J. (2003). Compositional Variations in Aged and Heated Pistacia Resin Found in Late Bronze Age Canaanite Amphorae and Bowls from Amarna, Egypt. Archaeometry.

[44] Tushingham S., Snyder C.M., Brownstein K.J., Damitio W.J., Gang D.R. (2018). Biomolecular Archaeology Reveals Ancient Origins of Indigenous Tobacco Smoking in North American Plateau. Proc. Natl. Acad. Sci. USA.

[45] Bailly L., Adam P., Charrié A., Connan J. (2016). Identification of Alkyl Guaiacyl Dehydroabietates as Novel Markers of Wood Tar from Pinaceae in Archaeological Samples. Org. Geochem..

[46] Charrié-Duhaut A., Connan J., Rouquette N., Adam P., Barbotin C., de Rozières M.-F., Tchapla A., Albrecht P. (2007). The Canopic Jars of Rameses II: Real Use Revealed by Molecular Study of Organic Residues. J. Archaeol. Sci..

[47] Colombini M.P., Modugno F., Silvano F., Onor M. (2000). Characterization of the Balm of an Egyptian Mummy from the Seventh Century B.C. Stud. Conserv..

[48] Jones J., Higham T.F.G., Oldfield R., O’Connor T.P., Buckley S.A. (2014). Evidence for Prehistoric Origins of Egyptian Mummification in Late Neolithic Burials. PLoS ONE.

[49] Łucejko J., Connan J., Orsini S., Ribechini E., Modugno F. (2017). Chemical Analyses of Egyptian Mummification Balms and Organic Residues from Storage Jars Dated from the Old Kingdom to the Copto-Byzantine Period. J. Archaeol. Sci..

[50] Saab A.M., Harb F.Y., Koenig W.A. (2005). Essential Oil Components in Heart Wood of Cedrus Libani and Cedrus Atlantica from Lebanon. Minerva Biotecnol..

[51] Ainane T. (2018). Chemical Characterization on the Aromatic Composition of Cedrus Atlantica from Morocco in Two Geographical Areas Will Break. AOICS.

[52] Chaudhary A., Kaur P., Singh B., Pathania V. (2009). Chemical Composition of Hydrodistilled and Solvent Volatiles Extracted from Woodchips of Himalayan Cedrus: Cedrus Deodara (Roxb.) Loud. Nat. Prod. Commun..

[53] Oukhrib A., Zaki M., Benharref A. (2018). The Chemistry of the Himachalenes and Atlantones from Cedrus. Arkivoc.

[54] Singh D., Agarwal S.K. (1988). Himachalol and?-Himachalene: Insecticidal Principles of Himalayan Cedarwood Oil. J. Chem. Ecol..

[55] Ottensmann M., Stoffel M.A., Nichols H.J., Hoffman J.I. (2018). GCalignR: An R Package for Aligning Gas-Chromatography Data for Ecological and Evolutionary Studies. PLoS ONE.

[56] R Core Team R: A Language and Environment for Statistical Computing.

