# Supplementary material for: Chemical Modification of Biomarkers through Accelerated Degradation: Implications for Ancient Plant Identification in Archaeo-Organic Residues
Source: Molecules. 2022 May 22;27(10):3331. doi: 10.3390/molecules27103331 (PMC9145360; doi:10.3390/molecules27103331)
Supplement: Supplementary file 1 [file molecules-27-03331-s001.zip › Supplementary material_FigureS1.pdf]

Figure S1: Principal component analysis (PCA) plots where the first four principal components (PC) are paired against each other for each of the three compound groups (G1, G2 and G3). The percentages of each PC indicate the variability explained by each PC axes.

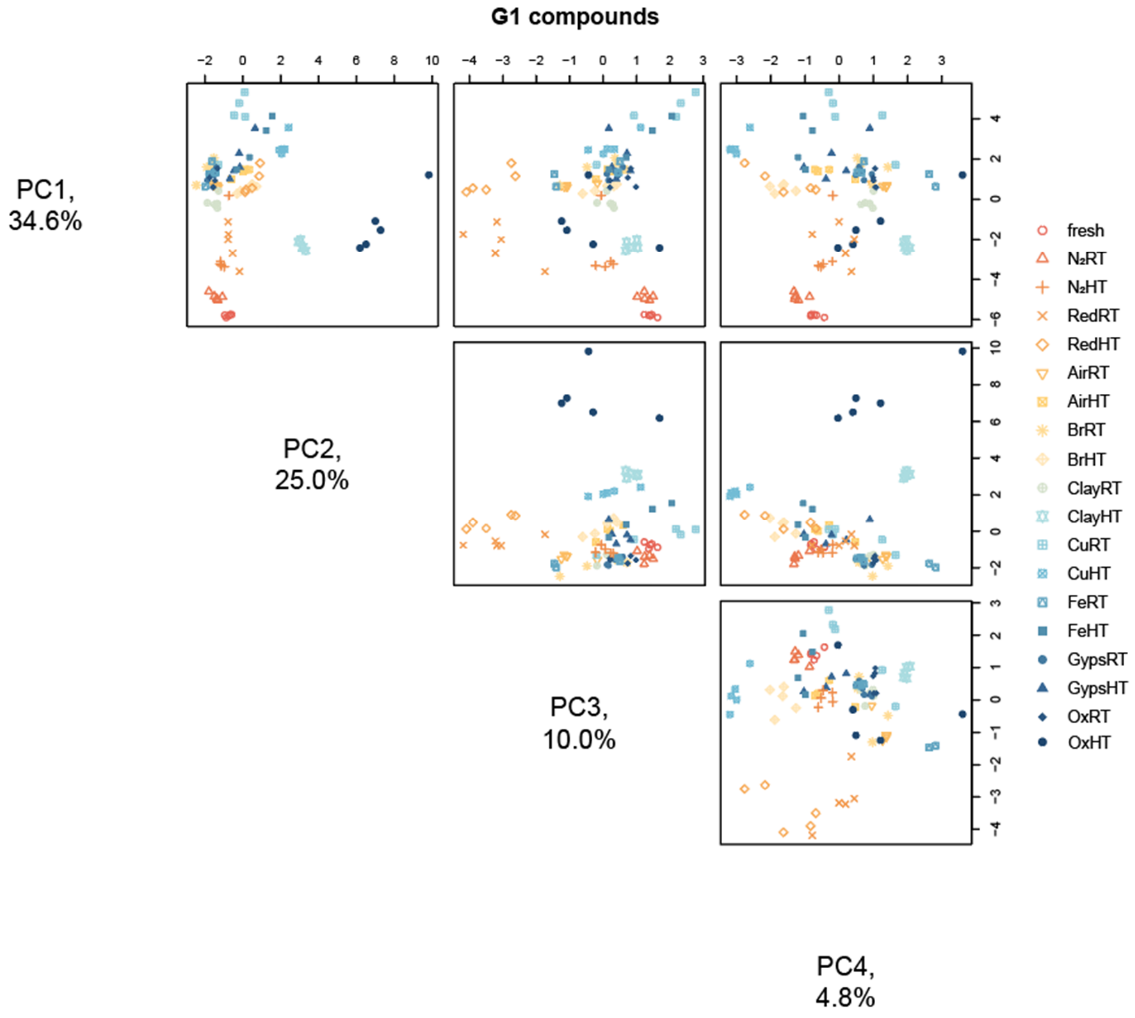

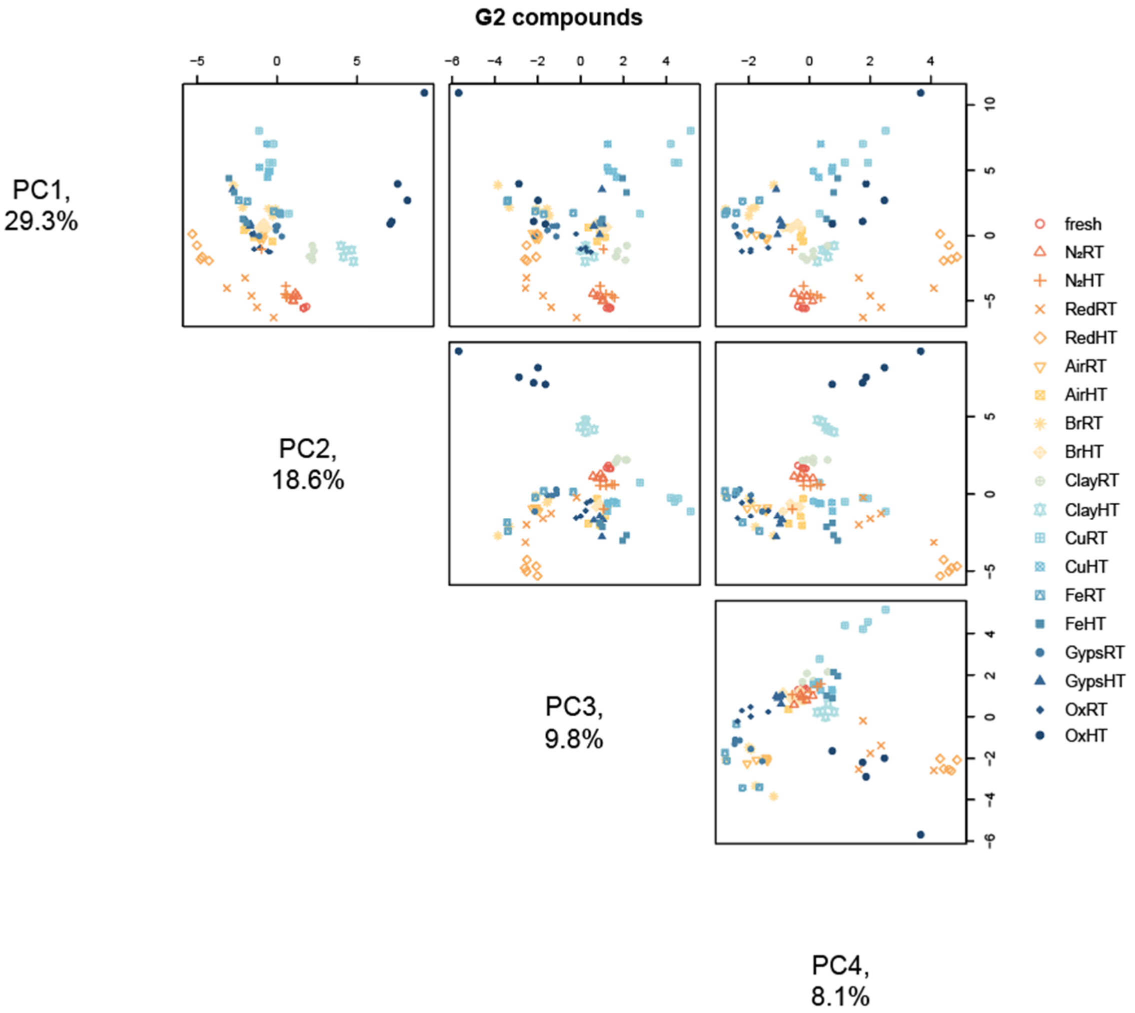

G2 compounds
PC1,
29.3%
PC2,
18.6%
PC3,
9.8%
PC4,
8.1%
fresh
N₂RT
N₂HT
RedRT
RedHT
AirRT
AirHT
BrRT
BrHT
ClayRT
ClayHT
CuRT
CuHT
FeRT
FeHT
GypsRT
GypsHT
OxRT
OxHT

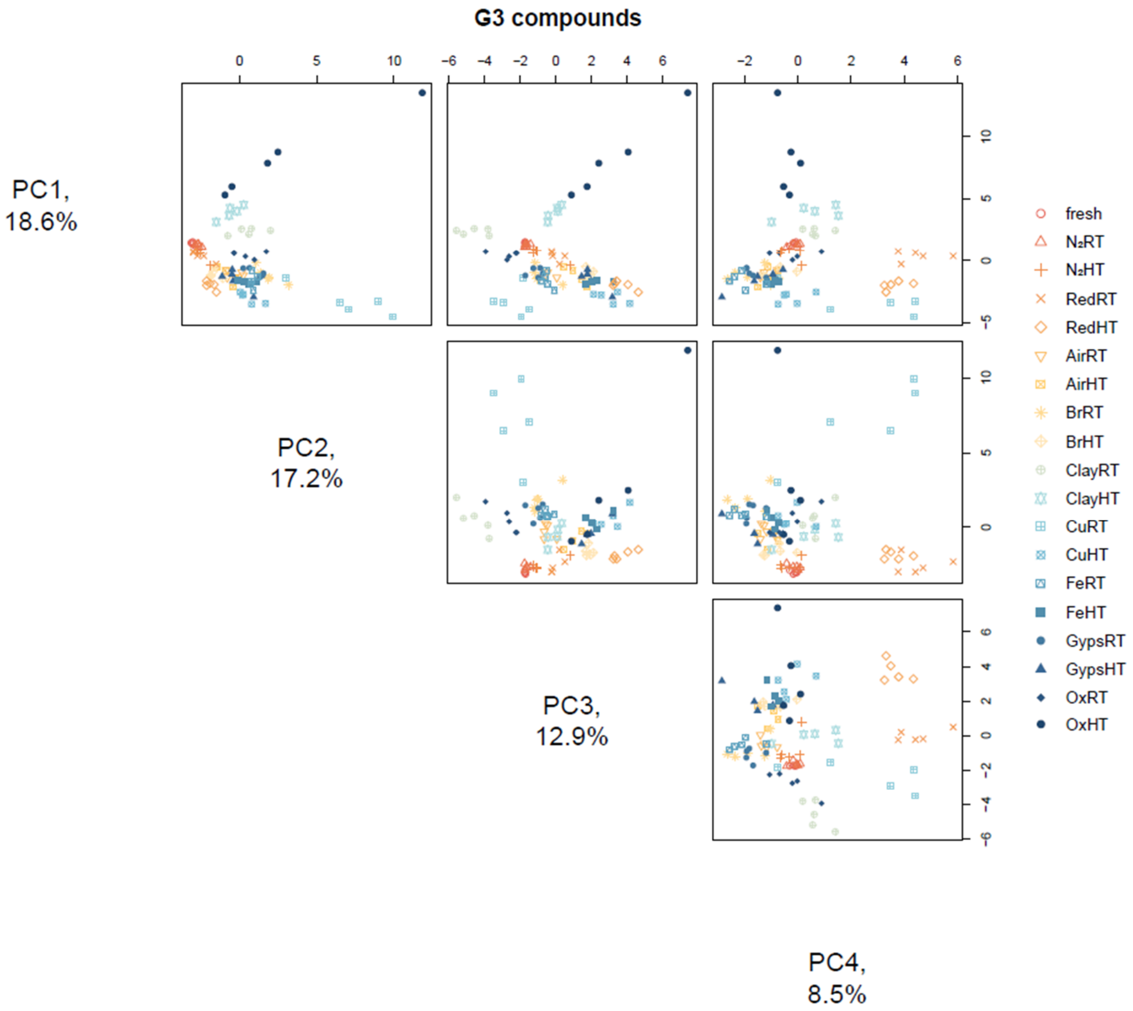
G3 compounds
PC1, 18.6%
PC2, 17.2%
PC3, 12.9%
PC4, 8.5%
fresh
N2RT
N2HT
RedRT
RedHT
AirRT
AirHT
BrRT
BrHT
ClayRT
ClayHT
CuRT
CuHT
FeRT
FeHT
GypsRT
GypsHT
OxRT
OxHT
